# Occurrence and Genotypic Identification of *Blastocystis* spp. and *Enterocytozoon bieneusi* in Bamaxiang Pigs in Bama Yao Autonomous County of Guangxi Province, China

**DOI:** 10.3390/ani14223344

**Published:** 2024-11-20

**Authors:** Xingang Yu, Xuanru Mu, Kaijian Yuan, Sifan Wang, Yilong Li, Hui Xu, Qiaoyu Li, Wenjing Zeng, Zhili Li, Jianchao Guo, Yang Hong

**Affiliations:** 1 School of Animal Science and Technology, Foshan University, Foshan 528231, China; yuxingang4525@163.com (X.Y.); 15940815092@163.com (X.M.); yuankj0195@163.com (K.Y.); 20230420301@stu.fosu.edu.cn (S.W.); muylyl20001110@163.com (Y.L.); 2112359031@stu.fosu.edu.cn (H.X.); 2112459167@stu.fosu.edu.cn (Q.L.); 2112459159@stu.fosu.edu.cn (W.Z.); pinganzhili@163.com (Z.L.); 2 Agro-Tech Extension Center of Guangdong Province, Guangzhou 510500, China; 3 National Key Laboratory of Intelligent Tracking and Forecasting for Infectious Diseases, National Institute of Parasitic Diseases, Chinese Center for Disease Control and Prevention (Chinese Center for Tropical Diseases Research), National Health Commission of the People’s Republic of China (NHC) Key Laboratory of Parasite and Vector Biology, World Health Organization (WHO) Collaborating Center for Tropical Diseases, National Center for International Research on Tropical Diseases, Shanghai 200025, China; 4 Hainan Tropical Diseases Research Center (Hainan Sub-Center, Chinese Center for Tropical Diseases Research), Haikou 571199, China

**Keywords:** *Blastocystis* spp., *Enterocytozoon bieneusi*, Bamaxiang pig, zoonotic, genotype

## Abstract

*Blastocystis* spp. and *Enterocytozoon bieneusi* are common intestinal pathogens capable of infecting both humans and animals, which lead to severe diarrhea and other intestinal diseases, posing a threat to public health. The Bamaxiang pig, a specialty of Bama Yao Autonomous County in Guangxi Province, China, is an important local breed in the regional pork market and an excellent model animal for biomedical research. Currently, no data is available on the prevalence or genotype distribution of these pathogens in Bamaxiang pigs. This study aimed to determine the prevalence and genetic characteristics of *Blastocystis* spp. and *E. bieneusi* in three Bamaxiang pig farms located in Bama Yao Autonomous County, using molecular techniques based on the small subunit ribosomal RNA (*SSU* rRNA) gene fragment of *Blastocystis* spp. and the internal transcribed spacer (ITS) region of *E. bieneusi*. All positive PCR products from the 311 fecal samples were sequenced to identify the species and genotypes of these organisms. The overall infection rates of *Blastocystis* spp. and *E. bieneusi* were 34.08% (106/311) and 18.32% (57/311), respectively. Three subtypes of *Blastocystis* spp. were detected: ST1 (*n* = 8), ST3 (*n* = 3), and ST5 (*n* = 95). Among them, zoonotic ST5 was the dominant genotype, accounting for 89.62% (95/106) of strains, followed by the genotypes ST1 (7.54%, 8/106) and ST3 (2.83%, 3/106). Two genotypes of *E. bieneusi* were detected: EbpC (*n* = 52) and CHG23 (*n* = 5), with EbpC being the dominant genotype. The human-pathogenic subtypes (ST1, ST3, and ST5) and genotypes (EbpC, CHG23) that were observed in this study indicate a potential threat to public health. Our findings offer basic information for preventing and controlling these zoonotic pathogens in the study area. Additional investigations are necessary to better understand their genetic characteristics and zoonotic potential within Guangxi Province.

## Simple Summary

The Bamaxiang pig is a distinctive breed native to Bama Yao Autonomous County in Guangxi Province, China. It plays a significant role in the regional pork market, is a popular companion animal, and is used as an animal model in scientific research. In this study, we report the occurrence and genetic characteristics of *Blastocystis* spp. and *Enterocytozoon bieneusi* in Bamaxiang pigs from Guangxi Province. The overall prevalence rates for *Blastocystis* spp. and *E. bieneusi* were found to be 34.08% (106/311) and 18.32% (57/311), respectively. We identified three subtypes of *Blastocystis* spp. (ST1, ST3, and ST5) and two genotypes of *E. bieneusi* (EbpC and CHG23), all of which are recognized as zoonotic genotypes. This is the first report on the occurrence and genetic characteristics of *Blastocystis* spp. and *E. bieneusi* in pigs from Guangxi Province. The presence of these zoonotic pathogens in Bamaxiang pigs suggests their potential role in the transmission of zoonotic parasitic diseases, highlighting the importance of monitoring these infections in both livestock and human populations.

## 1. Introduction

*Blastocystis* spp. and *Enterocytozoon bieneusi* are two common zoonotic intestinal pathogens that cause diarrhea and enteric diseases in humans, companion animals, domestic animals, and wildlife [1]. The cysts or spores of both pathogens can persist for extended periods in the environment. Infections in humans and animals commonly result from fecal-oral transmission or exposure to contaminated water and food. These two pathogens generally result in asymptomatic infections when present in low quantities within the host; however, they can cause severe abdominal pain, diarrhea, lethargy, emaciation, and even death in extreme cases, leading to considerable public health issues and economic losses [2,3,4].

Molecular epidemiological studies have confirmed four primary microsporidial species that infect humans: *Enterocytozoon bieneusi*, *Encephalitozoon cuniculi*, *Encephalitozoon intestinalis*, and *Encephalitozoon hellem*. *Enterocytozoon bieneusi* is the most prevalent species, accounting for over 90% of zoonotic microsporidiosis cases, which can lead to life-threatening diarrhea in immunocompromised individuals, including those with AIDS or transplant recipients [5,6,7]. *E. bieneusi* infections in humans have been reported globally, with infection rates varying between 1.4% and 78% [8]. Based on the sequence of the ribosomal RNA (rRNA) genes, *E. bieneusi* is a complex species divided into 15 distinct genetic groups [9]. The first two groups (Groups 1 and 2) represent a significant proportion (94%) of the total genotypes and encompass the most known zoonotic genotypes. The remaining groups (3–15) primarily consist of host-adapted genotypes associated with specific animal species [9,10]. The global prevalence of *E. bieneusi* is 37.6% in domestic pigs and 8.1% in wild boars, with the EbpA and EbpC genotypes being the most commonly reported [11,12]. Previous studies have identified several zoonotic *E. bieneusi* genotypes in pigs in China, suggesting that pigs may act as dispersing agents and potential sources of human infections [13,14]. 

*Blastocystis* exhibits considerable genetic diversity, with over 40 subtypes (STs) identified in humans and animals [15,16,17]. Among the approved subtypes of *Blastocystis* species, ST1 to ST10, ST12 to ST14, ST16, ST23, ST35, and ST41 have been documented in humans [15,18]. *Blastocystis* infection in pigs has been reported worldwide, with an overall incidence rate of 52.4%. This infection encompasses eight zoonotic subtypes (ST1–ST7, ST10) and one subtype specifically adapted to pigs or suids (ST15), with ST5 being the dominant subtype [19]. In China, research on pigs has revealed the presence of ST1, ST3, ST5, and ST10, with ST5 also being the dominant subtype [20]. Given the presence of identical *Blastocystis* subtypes in humans and pigs and the similarity or exact match in their nucleic acid sequences, investigating the distribution of *Blastocystis* subtypes in reared pigs is crucial for preventing potential zoonotic infections in humans. 

**Figure 1:**
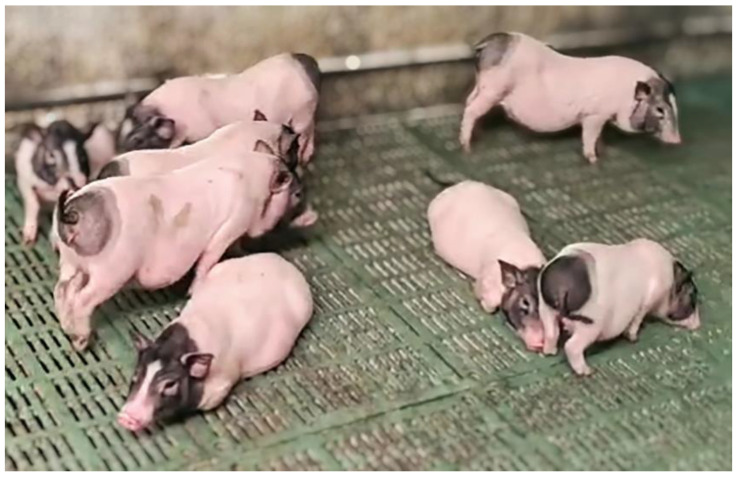
Bamaxiang pigs resting in the breeding farm.

The Bamaxiang pig is one of the most recognized indigenous purebred pig breeds in China. It is primarily found in Bama, Bailin, Natao, and Yantong counties in Guangxi province. This breed is known for its small body size and distinctive black coat with two ends (Figure 1). By 2005, the number of commercial Bamaxiang sows exceeded 100,000 [21]. Notably, the breed exhibits earlier sexual maturity, superior meat quality, and enhanced adaptability and disease resistance compared to other pig breeds [22]. Besides being a significant indigenous breed for the local pork market, the Bamaxiang pig also serves as an excellent animal model for biomedical research, demonstrating considerable potential in organ transplantation [23]. In recent years, Bamaxiang pigs have become popular as pet pigs, becoming beloved companions for people to enjoy and appreciate. However, there have been no reports of *Blastocystis* spp. and *E. bieneusi* infections in Bamaxiang pigs. Therefore, this study investigated the occurrence, genotypic distributions, and molecular characteristics of *Blastocystis* spp. and *E. bieneusi* in Bamaxiang pigs of different age groups across three large-scale pig farms in Guangxi Province, China.

## 2. Materials and Methods

### 2.1. Fecal Sample Collection

Between May 2024 and July 2024, 311 fresh fecal samples were collected from three farms of Bamaxiang pigs located in Bama Yao Autonomous County, Guangxi Province, China. These samples were from 70 suckling piglets (aged <21 days), 72 weaned piglets (aged 21–70 days), 78 fattening pigs (aged 71–180 days), and 91 sows (aged >180 days) [24]. Each fresh fecal sample was carefully placed into a labeled disposable plastic bag, which included information about the farm, pig age, and collection date. The samples were quickly transported on ice to the laboratory and stored in a 2.50% (*w*/*v*) potassium dichromate solution at 4 °C until they could be processed within a week.

### 2.2. DNA Extraction

Prior to DNA extraction, each fecal sample was washed with distilled water to remove any residues of potassium dichromate, ‘as’ previously described [25]. Approximately 200 mg of each fecal sample was processed for DNA extraction using the E.Z.N.A.^®®^ Stool DNA Kit (Omega Bio-tek Inc., Norcross, GA, USA), according to the manufacturer’s instructions. The fecal DNA samples were kept at −20 °C prior to polymerase chain reaction (PCR) analysis.

### 2.3. PCR Amplification

The DNA from all samples was amplified using PCR to detect either of the two target pathogens. *E. bieneusi* was detected through its ITS region [26], and *Blastocystis* spp. was identified by the small subunit (*SSU*) rRNA gene (Table 1) [27].

The PCRs were performed in 25 µL reaction systems: 8.5 µL nuclease-free deionized water, 12.5 µL 2× Taq Master Mix (Dye Plus) (Vazyme, Nanjing, China), 1 µL of each primer (10 µM each), and 2 µL genomic DNA for the primary PCR. The primary PCR product served as the secondary PCR. The reaction procedure for *E. bieneusi* was as follows: initial denaturation at 94 °C for five min, followed by 35 cycles of denaturation at 94 °C for 45 s, annealing at 57 °C for 30 s, extension at 72 °C for one min, and a final extension at 72 °C for 10 min. In the second round, the PCR product from the first round was used as the template, with annealing performed at 55 °C [25]. The amplification conditions for *Blastocystis* spp. consisted of an initial step at 94 °C for five min, followed by 35 cycles of one min at 94 °C, 45 s at 56 °C, and 45 s at 72 °C, with a final extension at 72 °C for 10 min [27].

The PCR products were visualized by electrophoresis on a 1.50% agarose gel stained with NA-Red (Beyotime, Nantong, China) and documented using the Azure™ c200 Gel Image Analysis System (Dublin, CA, USA).

### 2.4. Sequencing and Phylogenetic Analysis

All positive PCR products were sent to Sangon Biological Engineering Technology and Service Co., Ltd. (Songjiang, Shanghai, China) for DNA sequencing. The basic local alignment search tool (BLAST) was used to compare the sequences of *E. biseneusi* and *Blastocystis* spp. that were obtained (Files S1 and S2) with publicly available international reference sequences from the NCBI GenBank™ database (Tables S1 and S2), respectively. Phylogenetic trees of *E. biseneusi* and *Blastocystis* spp. were constructed using the maximum likelihood (ML) method in MEGA 7.0 (http://www.megasoftware.net/, accessed on 19 August 2024) to assess their genetic relationships. One thousand bootstrap replicates were performed to evaluate the robustness of the trees.

### 2.5. Statistical Analysis

The prevalence of *Blastocystis* spp. and *E. bieneusi* infections, including 95% confidence intervals (95% CI), were calculated. Statistical comparisons were conducted using Pearson’s chi-squared test (χ^2^) in crosstabs of SPSS version 27.0 (IBM SPSS Inc., Chicago, IL, USA) to analyze differences in prevalence between sample collection sites by age group. Results were considered significant if *p* < 0.05.

## 3. Results

### 3.1. Occurrence of Blastocystis *spp.* and E. bieneusi

A total of 106 *Blastocystis* spp. positive samples were detected, resulting in an overall infection rate of 34.08% (106/311; 95% CI: 28.8–39.4). The infection rates among different groups were as follows: suckling piglets (32.71%; 25/70; 95% CI: 27.2–47.2), weaned piglets (37.50%; 27/72; 95% CI: 26.0–49.0), fattening group (43.59%; 34/78; 95% CI: 32.3–54.8), and the sow group (21.98%; 20/91; 95% CI:13.3–30.6), and the differences were statistically significant (*p* < 0.05). The fattening group had the highest prevalence (Table 2) among the different growing stages of pigs. Fattening pigs exhibited a 2.74-fold higher risk of *Blastocystis* infection (95% CI: 1.41–5.35) compared to sows.

The overall infection rate of *E. bieneusi* was 18.32% (57/311; 95% CI: 14.0–22.7). Among the different growth stages of pigs, the weaned piglet group showed the highest infection prevalence at 30.56% (22/72; 95% CI: 19.7–41.5), followed by fattening pigs with a prevalence of 25.64% (20/78; 95% CI: 15.7–35.5), while suckling piglets had a prevalence of 17.14% (12/70; 95% CI: 15.7–35.5). The risk of *E. bieneusi* infection in weaned piglets was 12.91 times higher (95% CI 3.68–45.29) than in sows. Significant differences in infection rates were observed among the different growth stages of the pigs under investigation (*p* < 0.001) (Table 2).

From the perspective of coinfection, the overall percentage of pigs infected with both pathogens was 15.75% (49/311). The percentages of pigs infected with only *Blastocystis* spp. and *E. bieneusi* were 18.32% (57/311) and 2.57% (8/311), respectively. Furthermore, a comparative analysis of *Blastocystis* spp. and *E. bieneusi* across the three farms was conducted respectively, with no statistically significant differences in the prevalence of either pathogen among the three farms (Table S3).

### 3.2. Distributions of Blastocystis *spp.* Subtypes

**Figure 2:**
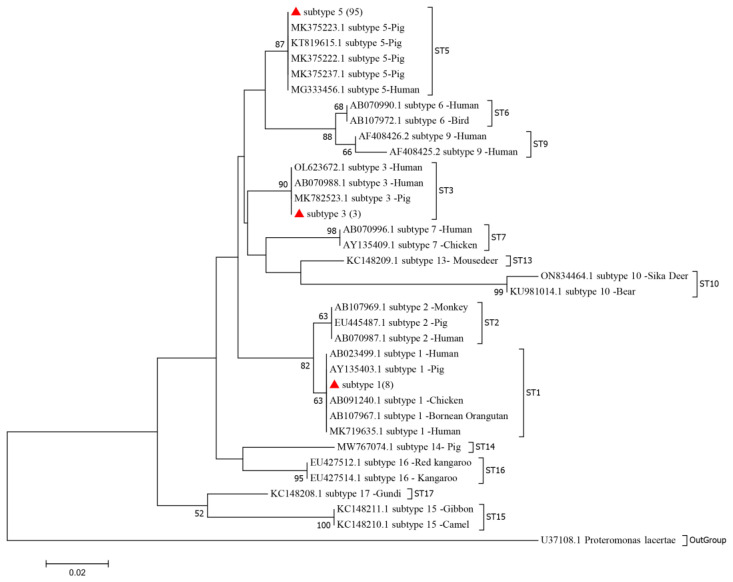
Phylogenetic analysis of *Blastocystis* spp. subtypes based on sequences of the *SSU* rRNA gene using the Tamura–Nei model method. A bootstrap algorithm was used to assess the branch reliability with 1000 replicates. Only bootstrap values above 50% are shown. Each sequence is identified by its accession number, genotype designation, and host origin. Sequences marked with red triangles indicate the sequences obtained in this study.

The sequencing analysis of 106 positive samples of *Blastocystis* spp. revealed three subtypes: ST1 (8/106), ST3 (3/106), and ST5 (95/106) (Table 2, Figure 2). ST5 was identified as the dominant and zoonotic genotype, accounting for 89.62% (95/106) of the positive samples, with 99% homology to the Yunnan porcine isolate. Notably, the ST1 and ST3 sequences obtained from pigs in this study are closely related to the ST1 sequence and the ST3 sequence derived from humans. Phylogenetic analysis indicated that the ST1, ST3, and ST5 sequences obtained from pigs in this study clustered into a clade with ST1, ST3, and ST5 sequences from other animals and humans.

### 3.3. Genotypes of E. bieneusi

**Figure 3:**
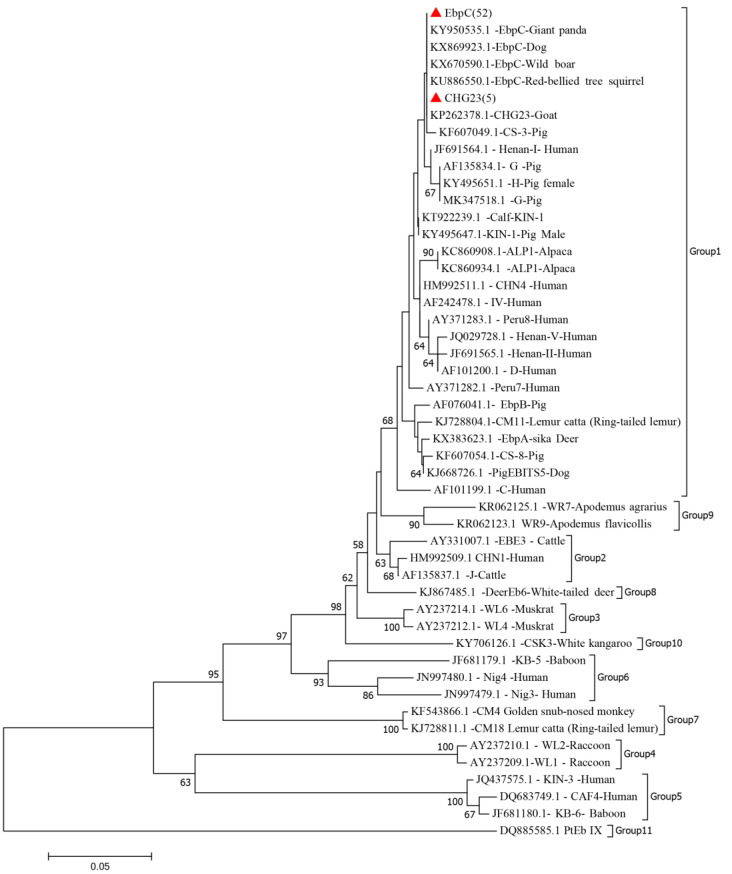
Phylogenetic relationship of *E. bieneusi* groups based on sequences of the ITS gene using the Tamura–Nei model method. A bootstrap algorithm was used to assess the branch reliability with 1000 replicates. Only bootstrap values above 55% are shown. Each sequence is identified by its accession number, genotype designation, and host origin. Sequences marked with red triangles indicate those obtained in this study.

In this study, 311 Bamaxiang pigs were tested, of which 57 were positive for PCR products of *E. bieneusi*. The sequences of the ITS region from these 57 positive samples were analyzed. The positive isolates were identified as subtypes EbpC and CHG23 (Table 2, Figure 3). Subtype EbpC was the dominant genotype in this study, accounting for 91.23% (52/57), with 100% homology to the gene from the Chinese giant panda. The five positive samples exhibited more than 99% homology with the CHG23 subtype derived from Chinese goats. Based on phylogenetic analyses of ITS sequences from this study and reference sequences downloaded from GenBank, all isolated genotypes were classified as group 1 with zoonotic characteristics.

## 4. Discussion

*Blastocystis* spp. are common intestinal protozoans that infect humans and various animals, including pigs, as well as other domestic and wild species. This protozoan often leads to symptoms such as diarrhea, weight loss, and decreased feeding efficiency. Globally, the prevalence of *Blastocystis* spp. in human populations has been reported to range from 22.0% to 56.0% in Europe and between 37.0% and 100.0% in various African and Asian countries [28]. In China, *Blastocystis* infections have been reported in humans in at least 12 provinces. The prevalence of these infections varies significantly, ranging from 0.007% to 48.6%, with an average infection rate of 3.37% [29]. Nevertheless, the overall infection rate of *Blastocystis* spp. in pigs was approximately 50.9% worldwide, with the prevalence in domestic pigs (52.4%) being higher than in wild pigs (31.2%) [19]. In this study, 106 out of 311 pig feces samples tested positive for *Blastocystis* spp., resulting in a total detection rate of 34.08% (106/311, 95% CI: 28.8–39.4). This result is similar to the current global rates of *Blastocystis* spp. infection in pigs. However, when compared to the prevalence reported in other studies, it is higher than the positive prevalence found in Heilongjiang (17.9%) [30], Hunan (22.89%) [24], Slovakia (12%) [31], and Jiangxi (30.50%) [24], but lower than that in Fujian (43.70%) [24], Anhui (43.2%) [32], the Thai-Myanmar border (37.2%) [33], and Shaanxi (74.8%) [34]. Several factors may contribute to the differences in infection rates. Variations in feeding management, sampling locations, sampling times, pig breeds, and hygienic conditions may affect the positivity rate of *Blastocystis* spp. infection. In addition, this study found that the infection rates for suckling piglets, weaned piglets, fattening pigs, and sows were 35.71% (95% CI: 27.2–47.2), 37.50% (95% CI: 26.0–49.0), 43.59% (95% CI: 32.3–54.8), and 21.98% (95% CI:13.3–30.6), respectively (Table 2). Previous reports indicated that the prevalence of *Blastocystis* sp. in sows was generally significantly higher than in fattening pigs [20]. However, in this study, the fattening group exhibited the highest infection rate of *Blastocystis*, consistent with the results of several earlier studies [24]. This difference may have resulted from the rearing conditions and feeding management. Interestingly, the suckling piglets exhibited the lowest infection rate apart from the sows group. This finding also agrees with the results of prior research [20,24], and the underlying reason may be related to the significant role of maternal antibodies.

*Blastocystis* infection in domesticated pigs has been documented worldwide, with nine subtypes (ST1–ST7, ST10, and ST15) reported in domestic pigs, while six subtypes (ST1, ST3–ST5, ST8, and ST15) have been isolated from wild boars. Prior research on pigs in specific regions of China has detected the presence of ST1, ST3, ST5, ST10, and mixed infections, with ST5 identified as the dominant subtype [20,35]. Based on genetic typing and a phylogenetic analysis of the *SSU* rRNA gene sequence in the present study, three subtypes of *Blastocystis* spp. were identified: ST1 (*n* = 8), ST3 (*n* = 3), and ST5 (*n* = 95). Among these, ST5 was the predominant subtype, representing 89.62% (95/106) of infections, which is consistent with the results from the majority of studies conducted in China. In Australia, close contact between pig herds and workers showed a high prevalence of the ST5 subtype, suggesting potential transmission between animals and humans [36]. A meta-analysis conducted in China revealed that human samples exhibited eight subtypes (ST1–ST7 and ST12), with ST1–ST3 being the most common. Notably, in patients with diarrhea, ST1 was the predominant subtype, while ST3 was the most frequently observed in asymptomatic cases [37]. *Blastocystis* ST1 is recognized as a pathogenic subtype linked to irritable bowel syndrome diarrhea (IBS-D). Similarly, *Blastocystis* ST3 is viewed as virulent, enhancing the parasite’s pathogenicity and raising serum IgE levels, which can lead to allergic reactions [37,38]. In this study, all 106 positive samples were identified as zoonotic genotypes, indicating the need for improved management measures in Bamaxiang pig farms. The presence of these zoonotic genotypes poses a risk of transmission to humans and other animals, underscoring their potential threat. Consequently, individuals who work long-term in pig farms, slaughterhouses, or reside near water bodies should prioritize personal protective measures and dietary hygiene. Further epidemiological investigations on *Blastocystis* spp. among the local population and water sources are needed while remaining vigilant to the potential public health issues posed by porcine-derived *Blastocystis* spp.

*E. bieneusi* is one of the most important causative agents of microsporidiosis, leading to diarrhea and other enteric disease symptoms in both humans and animals [39]. Reports of *E. bieneusi* in pigs are increasing globally, with the majority of cases in China reported from the central, northern, northeastern, eastern, and southeastern regions [9]. However, to date, there have been no reports of *E. bieneusi* in pig populations in Guangxi Province, located in the southwestern region of China. This is the first report of *E. bieneusi* in Bamaxiang pigs in Guangxi Province, with a prevalence of 18.32% (57/311; 95% CI: 13.0−23.8%), which is lower than the prevalence reported in most provinces or autonomous areas in China, such as Shaanxi (79.8%), Heilongjiang (55.7%), Henan (54.2%), Xinjiang (48.6%), Hainan (46.8%), Jilin (43.9%), Tibet (43.2%), Zhejiang (37.9%), Inner Mongolia (37.5%), Sichuan (31.2%), Yunnan (29.5%), Guangdong (26.4%), Liaoning (23.2%), and Fujian (21.5%), but higher than in Hunan (1.2%) and Jiangxi (6.1%) [9,12]. When comparing the findings with those from other countries in Asia, the infection rate exceeded the reported rates in Japan (13.7%), Thailand (14.0%), and Korea (14.2%) but was lower than in Malaysia (34.9%) [40]. These differences may also be partially attributed to variations in geoecology, seasonal factors, detection methods, the age of the pigs, and overall animal management.

The molecular characterization identified only two *E. bieneusi* genotypes: EbpC (*n* = 52) and CHG23 (*n* = 5). EbpC was the dominant genotype, accounting for 91.23% of the positive samples in the present study, consistent with the results of previous studies [9,12,41]. To date, in studies of pig herds, the genotype EbpC has been documented in at least 14 countries and across 14 provinces or autonomous regions in China [9,12]. Furthermore, EbpC, along with types IV and D, which belong to the largest genetic group, Group 1, exhibits the broadest host and geographic distributions. Notably, these types are also the most frequently identified in human populations [42]. Yang et al. [8] found that genotype EbpC was the most common among hospitalized and primary school children in the tested samples while also identifying the co-occurrence of genotypes CS-4, EbpC, and Henan-IV in both children and pigs in the same study area, suggesting that pigs could be a significant source of human *E. bieneusi* infections in northeast China. Meanwhile, genotype EbpC has also been occasionally detected in wastewater in Wuhan and Qingdao, China [8,43]. Remarkably, the CHG23 genotype, which is typically rarely detected, was identified in this study. Previously, it had only been reported in pigs from Fujian Province [44] and in goats from Henan Province [45]. Although rarely observed in pig populations, phylogenetic analysis revealed that CHG23, similar to genotype EbpC, belongs to Group 1, which is characterized by its zoonotic potential. This finding merits careful attention due to the potential of these genotypes to cause *E. bieneusi*-related microsporidiosis in humans.

Bama Yao Autonomous County, located in the northwest of Guangxi Province, features a subtropical climate characterized by typical Karst plateau topography. The region experiences an average annual relative humidity of 78%, with occasional flood events that facilitate the proliferation of various microbial species [46]. Additionally, environmental shedding of *Blastocystis* spp. and *E. bieneusi* spores by pigs from large-scale factory farms could lead to groundwater and surface water contamination during the rainy season. Moreover, Bama County is predominantly inhabited by ethnic minorities who frequently consume raw foods and homemade fruit wines, where large quantities of rice noodles and other ingredients are often immersed in water, rendering them susceptible to contamination by *Blastocystis* and *E. bieneusi*. Strengthening manure management on farms and enhancing public health awareness among villagers is crucial for preventing and controlling these zoonotic pathogens.

## 5. Conclusions

This study determined the prevalence rates of *Blastocystis* spp. (34.08%) and *E. beneusi* (18.32%) in three large-scale Bamaxiang pig farms in Guangxi Province. The subtypes ST1, ST3, and ST5 isolated from *Blastocystis* spp., as well as the subtypes EbpC and CHG23 identified from *E. bieneusi*, are all zoonotic. Our findings provided valuable insights into the molecular epidemiology of *Blastocystis* spp. and *E. bieneusi* in Bamaxiang pigs. Further research with a broader regional focus is needed to understand the epidemiology and genotypic characteristics of these pathogens among animal husbandry workers and in local water sources.

## Figures and Tables

**Table 1:** Primers used in the characterization of the *E. bieneusi* and *Blastocystis* spp.

Parasite	Gene		Primer Sequences (5′-3′)	Annealing Temperature (°C)	Fragment Length (bp)	Reference
*E. biseneusi*	ITS	F1	GATGGTCATAGGGATGAAGAGCTT	57	392	[25]
		R1	TATGCTTAAGTCCAGGGAG			
		F2	AGGGATGAAGAGCTTCGGCTCTG	55		
		R2	AGTGATCCTGTATTAGGGATATT			
*Blastocystis* spp.	*SSU* rRNA	F	GGAGGTAGTGACAATAAAC	56	550–585	[27]
		R	TAAGACTACGAGGGTATCTA			

**Table 2:** Colonization frequency and genotypes of *Blastocystis* spp. and *E. bieneusi* indifferent age groups.

Age (Months)	Sample Size (*n*)	*Blastocystis*					*E. bieneusi*				
		No. Positive	Subtypes (*n*)	Prevalence % (95% CI)	OR (95% CI)	*p* Value	No. Positive	Subtypes (*n*)	Prevalence % (95% CI)	OR (95% CI)	*p* Value
Suckling piglets (<21 days)	70	25	ST5 (*n* = 24) ST1 (*n* = 1)	35.71% (27.2–47.2%)	1.97 (0.98–3.96)	<0.05	12	EbpC (*n* = 11) CHG23 (*n* = 1)	17.14% (8.1–26.2%)	6.07 (1.64–22.45)	<0.001
Weaned piglets (21–70 days)	72	27	ST5 (*n* = 22) ST1 (*n* = 3) ST3 (*n* = 2)	37.50% (26.0–49.0%)	2.13 (1.07–4.24)		22	EbpC (*n* = 19) CHG23 (*n* = 3)	30.56% (19.7–41.5%)	12.91 (3.68–45.29)	
Fattening pigs (71–180 days)	78	34	ST5 (*n* = 32) ST3 (*n* = 1) ST1 (*n* = 1)	43.59% (32.3–54.8%)	2.74 (1.41–5.35)		20	EbpC (*n* = 19) CHG23 (*n* = 1)	25.64% (15.7–35.5%)	10.12 (2.88–35.59)	
Sows (>180 days)	91	20	ST5 (*n* = 17) ST1 (*n* = 3)	21.98% (13.3–30.6%)	Reference		3	EbpC (*n* = 3)	3.30% (0.4–7.0%)	Reference	
Total	311	106	ST5 (*n* = 95) ST1 (*n* = 8) ST3 (*n* = 3)	34.08% (28.8–39.4%)			57	EbpC (*n* = 52) CHG23 (*n* = 5)	18.33% (14.0–221,222.7%)		

## Data Availability

All datasets are contained within the manuscript. Representative nucleic acid sequences reported in this paper have been submitted to NCBI GenBank under accession numbers PQ561063-PQ561168 (*Blastocystis* spp.) and PQ570607-PQ570663 (*E. bieneusi*).

## Supplementary Materials

The following supporting information can be downloaded at: https://www.mdpi.com/article/10.3390/ani14223344/s1. Table S1. GenBank accession numbers of all *SSU* rRNA gene reference sequences of *Blastocystis* spp. used for phylogenetic analysis. Table S2. GenBank accession numbers of all *ITS* gene reference sequences of *E. bieneusi* used for phylogenetic analysis. Table S3. Occurrence of *Blastocystis* spp. and *E. bieneusi* in Bamaxiang pigs in different farm groups. File S1. The raw sequences of the *SSU* rRNA gene of *Blastocystis* spp. used for phylogenetic analysis. File S2. The raw sequences of the *ITS* gene of *E. bieneusi* used for phylogenetic analysis.
