# Supplementary material for: Occurrence and Genotypic Identification of Blastocystis spp. and Enterocytozoon bieneusi in Bamaxiang Pigs in Bama Yao Autonomous County of Guangxi Province, China
Source: Animals (Basel). 2024 Nov 20;14(22):3344. doi: 10.3390/ani14223344 (PMC11591291; doi:10.3390/ani14223344)
Supplement: Supplementary file 1 [file animals-14-03344-s001.zip › Table S1. GenBank accession numbers of all SSU rRNA gene reference sequences of Blastocystis spp. used for phylogenetic analysis(1).pdf]

Table S1. GenBank accession numbers of all *SSU rRNA* gene sequences of *Blastocystis* spp. used for phylogenetic analysis (Figure 2), and associated information.

GenBank ID	Subtypes	Origin	Country	Genotype
MK375223.1	ST5	Pig	China	ST5
KT819615.1	ST5	Pig	Thailand	ST5
MK375222.1	ST5	Pig	China	ST5
MK375237.1	ST5	Pig	China	ST5
MG333456.1	ST5	Human	Thailand	ST5
AB070990.1	ST6	Human	Tokyo	ST6
AB107972.1	ST6	Bird	Osaka	ST6
AF408426.2	ST9	Human	Japan	ST9
AF408425.2	ST9	Human	Japan	ST9
OL623672.1	ST3	Human	Spain	ST3
AB070988.1	ST3	Human	Japan	ST3
MK782523.1	ST3	Pig		ST3
AB070996.1	ST7	Human	Japan	ST7
AY135409.1	ST7	Chicken	France	ST7
KC148209.1	ST13	Mousedeer	UK	ST13
ON834464.1	ST10	Bear	China	ST10
KU981014.1	ST10	Sika deer	Malaysia	ST10
AB107969.1	ST2	Monkey	Japan	ST2
EU445487.1	ST2	Pig	Philippines	ST2
AB070987.1	ST2	Human	Japan	ST2
AB023499.1	ST1	Human	Japan	ST1
AY135403.1	ST1	Pig	France	ST1
AB091240.1	ST1	Chicken	Japan	ST1
AB107967.1	ST1	Bornean orangutan	Japan	ST1
MK719635.1	ST1	Human	India	ST1
MW767074.1	ST14	Pig	China	ST14
EU427512.1	ST16	Red kangaroo	Japan	ST16
EU427514.1	ST16	Kangaroo	Japan	ST16
KC148208.1	ST17	Gundi	UK	ST17
KC148211.1	ST15	Gibbon	UK	ST15
KC148210.1	ST15	Camel	UK	ST15
U37108.1	not available	*Proteromonas lacertae*	USA	OutGroup
