# Supplementary material for: Occurrence and Genotypic Identification of Blastocystis spp. and Enterocytozoon bieneusi in Bamaxiang Pigs in Bama Yao Autonomous County of Guangxi Province, China
Source: Animals (Basel). 2024 Nov 20;14(22):3344. doi: 10.3390/ani14223344 (PMC11591291; doi:10.3390/ani14223344)
Supplement: Supplementary file 1 [file animals-14-03344-s001.zip › Table S2. GenBank accession numbers of all ITS gene reference sequences of E. bieneusi used for phylogenetic analysis.pdf]

Table S2. GenBank accession numbers of all ITS gene sequences of *E. bieneusi* used for phylogenetic analysis (Figure 3), and associated information.

GenBank ID	Subtypes	Origin	Country	Group
KY950535.1	EbpC	Giant panda	China	Group1
KX869923.1	EbpC	Dog	China	Group1
KX670590.1	EbpC	Wild boar	China	Group1
KU886550.1	EbpC	Red-bellied tree squirrel	China	Group1
KP262378.1	CHG23	Goat	China	Group1
KF607049.1	CS-3	Pig	China	Group1
JF691564.1	Henan- Ⅰ	Human	China	Group1
AF135834.1	G	Pig	Germany	Group1
KY495651.1	H	Pig femal	China	Group1
MK347518.1	G	Pig	China	Group1
KT922239.1	KIN-1	Calf	China	Group1
KY495647.1	KIN-1	Pig Male	China	Group1
KC860908.1	ALP1	Alpaca	Peru	Group1
KC860934.1	ALP1	Alpaca	Peru	Group1
AF242478.1	Ⅳ	Human	France	Group1
HM992511.1	CHN4	Human	China	Group1
AY371283.1	Peru8	Human	USA	Group1
JQ029728.1	Henan-Ⅴ	Human	China	Group1
JF691565.1	Henan- Ⅱ	Human	China	Group1
AF101200.1	D	Human	Germany	Group1
AY371282.1	Peru7	Human	Peru	Group1
AF076041.1	EbpB	Pig	Switzerland	Group1
KJ728804.1	CM11	Lemur cattle	China	Group1
KX383623.1	EbpA	Sika Deer	China	Group1
KF607054.1	CS-8	Pig	China	Group1
KJ668726.1	PigEBITS5	Dog	China	Group1
AF101199.1	C	Human	Germany	Group1
KR062125.1	WR7	Apodemus agrarius	Poland	Group9
KR062123.1	WR9	Apodemus flavicollis	Poland	Group9
AY331007.1	EBE3	Cattle	USA	Group2
HM992509.1	CHN1	Human	China	Group2
AF135837.1	J	Cattle	Germany	Group2
KJ867485.1	DeerEb6	white-tailed deer	USA	Group8
AY237214.1	WL6	Muskrat	USA	Group3
AY237212.1	WL4	Muskrat	USA	Group3
KY706126.1	CSK3	White kangaroo	China	Group10
JF681179.1	KB-5	Baboon	USA	Group6
JN997480.1	Nig4	Human	Nigeria	Group6

JN997479.1	Nig3	Human	Nigeria	Group6
KF543866.1	CM4	Golden snub monkey	China	Group7
KJ728811.1	CM18	Lemur cattle	China	Group7
AY237210.1	WL2	Raccoon	USA	Group4
AY237209.1	WL1	Raccoon	USA	Group4
JQ437575.1	KIN-3	Human	France	Group5
DQ683749.1	CAF4	Human	France	Group5
JF681180.1	KB-6	Baboon	Kenya	Group5
DQ885585.1	PtEb-IX	Dog	Portugal	OutGroup（Group11）
