# Supplementary material for: Occurrence and Genotypic Identification of Blastocystis spp. and Enterocytozoon bieneusi in Bamaxiang Pigs in Bama Yao Autonomous County of Guangxi Province, China
Source: Animals (Basel). 2024 Nov 20;14(22):3344. doi: 10.3390/ani14223344 (PMC11591291; doi:10.3390/ani14223344)
Supplement: Supplementary file 1 [file animals-14-03344-s001.zip › Table S3. Occurrence of Blastocystis spp. and E. bieneusi in Bamaxiang pigs in different farm groups 2024-11-9 YXG A1(1).pdf]

Table S3. Occurrence of *Blastocystis* spp. and *E. bieneusi* in Bamaxiang pigs in different farm groups.

Farm	Sample Size (n)	*Blastocystis*				*E. bieneusi*			
		**No. Positive**	**Subtypes (n)**	**Prevalence % (95% CI)**	***P* Value**	**No. Positive**	**Subtypes (n)**	**Prevalence % (95% CI)**	***P* Value**
Farm 1	103	42	ST5(n=38)<br>ST1(n=3)<br>ST3(n=1)	40.78%<br>(31.1%-50.4%)	0.188	19	EbpC(n=15)<br>CHG23(n=4)	18.45%<br>(10.8%-26.1%)	0.717
Farm 2	100	29	ST5(27)<br>ST1(n=1)<br>ST3(n=1)	29.00%<br>(20.0%-38.0%)		16	EbpC(n=16)	16.00%<br>(8.7%-23.3%)	
Farm 3	108	35	ST5(n=30)<br>ST1(n=4)<br>ST3(n=1)	32.40%<br>(23.4%-41.4%)		22	EbpC(n=21)<br>CHG23(n=1)	20.37%<br>(12.7%-28.1%)	
Total	311	106	ST5(n=95)<br>ST1(n=8)<br>ST3(n=3)	34.08%<br>(28.8%-39.4%)		57	EbpC(n=52)<br>CHG23(n=5)	18.33%<br>(14.0%-22.7%)	
